# Association Between Anxiety, Depression, and Post-traumatic Stress Disorder and Outcomes After Ischemic Stroke

**DOI:** 10.3389/fneur.2018.00890

**Published:** 2018-11-02

**Authors:** Laura A. Stein, Emily Goldmann, Ahmad Zamzam, Jean M. Luciano, Steven R. Messé, Brett L. Cucchiara, Scott E. Kasner, Michael T. Mullen

**Affiliations:** ^1^Department of Neurology, Perelman School of Medicine at the University of Pennsylvania, Philadelphia, PA, United States; ^2^College of Global Public Health, New York University, New York, NY, United States; ^3^Leonard Davis Institute of Health Economics, University of Pennsylvania, Philadelphia, PA, United States

**Keywords:** stroke, depression, anxiety, PTSD, mental health disorder

## Abstract

**Background:** Stroke patients are known to be at risk of developing anxiety, depression, and post-traumatic stress disorder (PTSD).

**Objective:** To determine the overlap between anxiety, depression, and PTSD in patients after stroke and to determine the association between these disorders and quality of life, functional status, healthcare utilization, and return to work.

**Methods:** A cross-sectional telephone survey was conducted to assess for depression, anxiety, PTSD, and health-related outcomes 6–12 months after first ischemic stroke in patients without prior psychiatric disease at a single stroke center.

**Results:** Of 352 eligible subjects, 55 (16%) completed surveys. Seven subjects (13%) met criteria for probable anxiety, 6 (11%) for PTSD, and 11 for depression (20%). Of the 13 subjects (24%) who met criteria for any of these disorders, 6 (46%) met criteria for more than one, and 5 (39%) met criteria for all three. There were no significant differences in baseline characteristics, including stroke severity or neurologic symptoms, between those with or without any of these disorders. Those who had any of these disorders were less likely to be independent in their activities of daily living (ADLs) (54 vs. 95%, *p* < 0.001) and reported significantly worse quality of life (score of 0–100, median score of 50 vs. 80, *p* < 0.001) compared to those with none of these disorders.

**Conclusions:** Anxiety, depression, and PTSD are common after stroke, have a high degree of co-occurrence, and are associated with worse outcomes, including quality of life and functional status.

## Introduction

Numerous studies have demonstrated that psychiatric symptoms are common after stroke (1). It has been postulated that the sudden onset of neurologic deficits may contribute to distress and anxiety beyond that seen with other acute medical illnesses (2, 3). Although depression has been most studied after stroke, there is an emerging literature on post-stroke anxiety and post-traumatic stress disorder (PTSD). These studies suggest that the post-stroke population has a prevalence of depression, anxiety, and PTSD, occurring in approximately one-quarter to one-third of patients (4–12).

Existing data outside of the stroke population suggest that mental health disorders have a significant impact on patient outcomes including quality of life and mortality (13). In the cardiac literature, depression has been linked to decreased quality of life, and increased all-cause mortality (14–17). Although stroke severity is a major driver of quality of life after stroke, some prior studies have shown reductions in quality of life that are out of proportion to neurologic deficits after stroke (18, 19). Given the relatively high prevalence of anxiety, depression, and PTSD after stroke, it is possible that these disorders are negatively impacting quality of life for these patients. Additionally, studies have linked depression to greater risk of recurrent stroke and death (20, 21).

Co-occurrence of these three mental health disorders is common in the general population, but this has not been well elucidated amongst stroke survivors (22). In one study of stroke patients, having more than one disorder was associated with greater odds of 6-month readmission or death (13). However, while prior studies have looked at outcomes, these studies have had limited data on stroke severity and/or neurologic deficits, which are major potential confounders (11, 23–26).

To that end, we sought to survey patients with first stroke and no reported history of prior psychiatric disease to determine the prevalence of anxiety, depression, and PTSD and the degree of overlap between these conditions. We additionally sought to identify demographic and clinical factors, including detailed information about stroke severity and neurologic deficits, associated with these disorders. Finally, we evaluated the association between these disorders and patient outcomes including return to work, healthcare utilization, self-reported functional outcome, and self-reported quality of life.

### Methods

A cross-sectional survey was conducted to assess for depression, anxiety, PTSD, and health outcomes 6–12 months after first ischemic stroke. The study population included all subjects with ischemic stroke discharged from the Comprehensive Stroke Center at the Hospital of the University of Pennsylvania between October 2014 and January 2016 who were age ≥ 18 and discharged to either home or acute inpatient rehabilitation. Patients who were discharged to skilled nursing facility or hospice were excluded, as were patients with prior known stroke, documented pre-existing psychiatric history (including generalized anxiety disorder, major depressive disorder, bipolar disorder, PTSD, panic disorder, schizophrenia, or schizoaffective disorder), severe aphasia, or cognitive disability which would prevent them from providing informed consent or completing the telephone-based survey. The Institutional Review Board at the University of Pennsylvania approved the study.

We identified potentially eligible subjects from the inpatient stroke quality—improvement database at our institution (Get with the Guidelines®-Stroke). Inpatient medical records were screened to identify potential exclusion criteria. Given evidence that post-stroke mental health disorders may be delayed after stroke, potentially eligible subjects were contacted by phone 6–12 months after hospital discharge (11, 25, 27). Three attempts were made to contact each potential subject. When contact was made, a standardized consent script was read to prospective subjects. Subjects who consented to participate had inclusion/exclusion criteria confirmed and were then administered a questionnaire using a standardized script that took ~20–30 min to complete (see Supplementary Material). Interviews were completed by the subject in all cases; caregivers were not permitted to provide answers.

We utilized the Hospital Anxiety and Depression (HADS) scale to assess for anxiety and depression. The HADS is a validated scale consisting of 14 questions, seven for depression and seven for anxiety (28–30). Each question has four responses, each response with a score of 0–3, with higher scores indicating greater frequency of symptoms. Scores across anxiety and depression are summed separately (range 0–21). A score of ≥8, on the anxiety or depression subset, defined those with depression or anxiety (29). The PTSD Checklist (PCL-S) was used to measure symptoms of PTSD. The PCL-S is a stressor specific PTSD checklist, based on Diagnostic and Statistical Manual of Mental Disorders 4th Edition (DSM-IV) criteria, consisting of 17 questions relating to the potentially traumatic event, in this case the subject's stroke (31). Item responses range from 1 (not at all) to 5 (extremely), with total scores ranging from 17–85. A score of ≥44 identified PTSD (31).

To measure outcomes, we used the Stanford Healthcare Utilization Survey to assess physician visits, emergency room visits, and inpatient admissions in the 6 months preceding survey administration (32). Functional status was assessed using the modified Rankin scale (mRS), which has been previously validated for telephone administration (33, 34). The mRS is a zero to five scale where zero equates to no symptoms and five indicates severe disability requiring 24-h care. For analytic purposes, mRS was dichotomized, with mRS 0–2 defined as a “good” outcome and mRS 3–5 a “bad” outcome. Quality of life was assessed with the EuroQOL EQ-5D. This validated questionnaire assesses five domains of overall health including mobility, self-care, usual activities, pain, and anxiety/depression (35, 36). Within each domain, subjects are asked whether they have no difficulty, some/moderate difficulty, or extreme difficulty. For analysis, these questions were dichotomized to no difficulty vs. any difficulty. Subjects were also asked to rate their overall health on a scale from 0 to 100 with 0 being the worst and 100 being the best overall health state.

For all potentially eligible subjects, age, sex, race, initial National Institutes of Health Stroke Scale score (NIHSS) (categorized as mild, moderate, and severe using NIHSS cut-points of 0–4, 5–9, and 10+, respectively), and discharge disposition (home vs. acute inpatient rehabilitation) were extracted from the quality-improvement database. Consented subjects were asked demographic information, level of education, employment status pre- and post-stroke, marital status, household size, acute rehabilitation stay duration, and whether they had a recurrent stroke. Subjects were asked about prior diagnosis of psychiatric disease to ensure they met eligibility criteria. From the medical record, we extracted details of the index stroke hospitalization, anatomic stroke location, and stroke mechanism using the modified TOAST criteria (large vessel atherosclerosis, small vessel infarction, cardioembolism, other determined etiology, and cryptogenic) (37). Physical exam data was retrospectively gathered based upon the last exam documented by the inpatient neurology team prior to discharge. For this analysis, weakness was graded as absent, mild, or moderate to severe (manual muscle testing score of 5/5, 4/5, and 0–3/5, respectively). Aphasia was rated as absent, mild, or moderate/severe. Dysarthria was rated as absent, mild/moderate, or severe. Sensory loss, ataxia, neglect, and vision loss were rated as present or absent. Additionally, gait distance was recorded from physical and occupational therapy notes. At our center, hospitalized stroke patients are assessed for probable depression using the 2-item Patient Health Questionnaire (PHQ-2). We recorded these inpatient PHQ-2 results for all subjects, and a score of 3 or greater was considered a positive screen (29).

### Statistical analysis

We calculated the total number of eligible subjects that we attempted to contact and the proportion of subjects who consented and completed the survey. Variables were summarized using means or medians as appropriate for continuous variables and frequencies and proportions for categorical variables. To assess for response bias, we compared enrolled subjects to eligible subjects who did not complete the survey on available demographic variables including age, sex, race, NIHSS, and discharge disposition. Because of the overlap between anxiety, depression, and PTSD, we elected to compare subjects who had any of these conditions to those who did not. Baseline and outcome variables were compared using Student's *t*-test or Wilcoxon rank sum test for continuous variables and χ^2^ or Fisher's exact tests (when any cell contained <5 observations) for categorical variables, as appropriate. We did not correct for multiple comparisons. Given the small sample size, multivariable regression was limited. In a *post-hoc* exploratory analysis we evaluated the association between the presence of anxiety, depression, and/or PTSD and quality of life, measured using the EuroQol visual analog scale, in multivariable linear regression including age, initial NIHSS, and mRS. Statistical analysis was conducted in STATA (StataCorp. 2017. Statistical Software Release 15, College Station, TX). Statistical significance was defined as *p* < 0.05.

## Results

**Figure 1:**
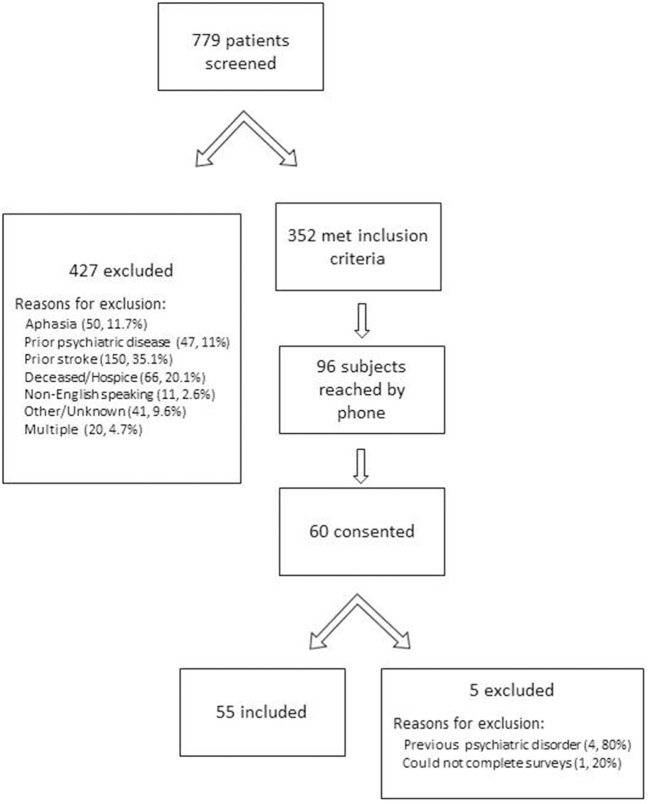
Screening of subjects.

There were a total of 779 patients screened, of which 352 were eligible for participation in the phone survey. Of those 352 potentially eligible subjects, 96 were successfully contacted, and 60 consented for participation. There were 5 subjects who were subsequently excluded after consenting because they confirmed a history of prior psychiatric disorder, prior stroke, or inability to complete the surveys, leaving 55 subjects who completed the survey (Figure 1).

Baseline demographic information from the quality-improvement database was collected on the 352 potentially eligible subjects, and the 55 enrolled subjects were compared to the 297 subjects who were not enrolled (Table 1). There were no significant differences between responders and non-responders with respect to age, sex, mean NIHSS, or discharge disposition. However, a greater proportion of those enrolled had a mild stroke (NIHSS 0–4 in 75% enrolled vs. 57% not enrolled, *p* = 0.03). The observed difference in race likely reflects incomplete documentation of race in the electronic medical record; enrolled patients were asked race directly.

**Table 1:** Baseline demographics of eligible subjects, comparing enrolled vs. not-enrolled.

	**Overall**	**Enrolled**	**Not enrolled**	***P*-value**
*n*	352	55	297	–
Age, mean	64 (15)	63 (12)	64 (15)	0.81^*^
Female sex	45%	42%	46%	0.62^**^
Race				0.02^†^
White	40%	51%	40%	
Black	40%	44%	39%	–
Asian/Pacific Islander	2%	4%	2%	–
Other/unknown	9%	2%	18%	–
Initial NIHSS, median (IQR)	3 (1–7)	3 (1–4)	3 (1–7)	0.18^‡^
Nihss Categories				0.03^†^
0–4	60%	75%	57%	
5–9	17%	7%	19%	–
10+	17%	16%	17%	–
Missing	7%	2%	7%	–
Discharge to home	55%	60%	54%	0.40^**^

*NIHSS, National Institutes of Health Stroke Scale; IQR, interquartile range*.

* *Student's t-test*.

** *Chi-squared test*.

† *Fisher's exact test*.

‡ *Wilcoxon Rank Sum*.

Of the 55 subjects who completed the survey, 7 (13%) had anxiety, 6 (11%) PTSD, and 11 (20%) depression. There was large overlap between these groups. Of the 7 subjects who had anxiety, 5 also had PTSD, and 5 had depression. All of the 6 subjects who had PTSD also had depression and 5 had anxiety. There were 5 subjects who had depression alone. Due to the high rates of overlap amongst these conditions, we combined all subjects who had anxiety, depression, or PTSD, into one group for the remainder of the analysis. In this sample, 24% of subjects met criteria for any of the three mental health disorders.

Baseline demographic characteristics of all enrolled subjects are described in Table 2. Comparing subjects with anxiety, depression and/or PTSD to those without, there were no statistically significant differences in baseline characteristics. However, a numerically higher proportion of subjects with one of these disorders did not complete high school compared to subjects without a mental health disorder. Stroke and in-hospital variables are summarized in Table 3. Overall, stroke severity was low (median NIHSS = 3 for all subjects) and there were no differences between groups in initial stroke severity, etiology, or stroke location. Although not statistically significant, more subjects with anxiety, depression, and/or PTSD went to inpatient rehabilitation (62 vs. 15%, *p* = 0.06), and length of stay (LOS) in inpatient rehabilitation tended to be longer in this group (median LOS 21 days vs. 14 days, *p* = 0.07). The PHQ-2 depression screen was conducted during the initial hospitalization in 45 of the 55 total subjects. It was positive during initial admission in 4 of 8 (50%) subjects with depression at 6–12 months and 4 of 10 (40%) of those who had anxiety, depression, and/or PTSD at 6–12 months. There were no significant differences in the type or severity of neurologic deficits at baseline between those with and without any mental disorder (Table 4).

**Table 2:** Baseline demographics for enrolled subjects comparing subjects meeting criteria for mental health disorders vs. subjects who do not.

		**Depression, anxiety, and/or PTSD**		***P*-value**
	**Overall**	**Absent**	**Present**	
*n*	55	42	13	–
Age, mean (SD)	63 (12)	64 (12)	62 (14)	0.74^*^
Female sex	42%	41%	46%	0.72^**^
Race				0.52^†^
White	51%	55%	39%	
Black	44%	38%	62%	–
Asian/Pacific Islander	4%	5%	0.0%	–
Other	2%	2%	0.0%	–
Hispanic ethnicity	2%	2%	0.0%	1.00^†^
Education				0.10^†^
Did not graduate HS	9%	5%	23%	
HS graduate	46%	52%	23%	–
Some college	29%	26%	39%	–
Grad/Prof degree	16%	17%	15%	–
Employed pre-stroke	58%	57%	62%	0.78^**^
Currently married	62%	62%	62%	0.98^**^
Household size, median (IQR)	2 (2, 4)	2 (2, 4)	3 (2, 4)	0.47^‡^
Days from stroke to survey, median (IQR)	276 (226, 320)	273 (226, 330)	278 (234, 295)	0.42^‡^

*PTSD, Post Traumatic Stress Disorder; SD, standard deviation; HS, high school; Grad, graduate degree; Prof, other professional degree; IQR, interquartile range*.

* *Student's t-test*.

** *Chi-squared test*.

† *Fisher's exact test*.

‡ *Wilcoxon Rank Sum*.

**Table 3:** Stroke Admission Characteristics among those with and without probable mental health disorder.

	**Overall**	**Depression, anxiety, and/or PTSD**		***P*-value**
		**Absent**	**Present**	
*n*	55	42	13	–
Nihss Categories				0.55^†^
0–4	75%	71%	85%	
5–9	7%	7%	8%	–
10+	18%	21%	8%	–
ICU stay	27%	31%	15%	0.48^†^
SSRI prescribed at discharge	7%	5%	15%	0.23^†^
Stroke Etiology At Discharge				0.93^†^
Large artery	18%	19%	15%	
atherosclerosis				
Small vessel disease	16%	14%	23%	–
Cardioembolism	12%	11%	15%	–
Other determined etiology	9%	10%	8%	–
Cryptogenic	44%	45%	39%	–
Stroke Territory				
ACA	0.0%	0.0%	0.0%	1.00^†^
MCA/ICA	73%	77%	62%	0.28^**^
PCA	10%	9%	15%	0.60^†^
Vertebral	6%	6%	8%	1.00^†^
Basilar	15%	17%	8%	0.66^†^
Stroke Location				
Cortical	38%	37%	39%	1.00^†^
Subcortical	67%	66%	69%	1.00^†^
Posterior	21%	20%	25%	0.70^†^
Discharge to acute rehab	38%	15%	62%	0.06^†^
Patient reported rehab duration, median, days (IQR)	21 (11–30)	14 (8–30)	21 (21–36)	0.07^‡^
Patient reported recurrent stroke	9%	7%	15%	0.58^†^

*PTSD, post traumatic stress disorder; NIHSS, National Institutes of Health Stroke Scale; ICU, intensive care unit; SSRI, selective serotonin reuptake inhibitor; ACA, anterior cerebral artery; MCA, middle cerebral artery; ICA, internal carotid artery; PCA, posterior cerebral artery; IQR, interquartile range*.

** *Chi-squared test*.

† *Fisher's exact test*.

‡ *Wilcoxon Rank Sum*.

**Table 4:** Neurologic deficits at hospital discharge.

	**Overall**	**Depression, anxiety, and/or PTSD**		***P*-value**
		**Absent**	**Present**	
*n*	55	42	13	–
Weakness (any)	58%	55%	69%	0.52^†^
Mod-severe weakness				
Manual Muscle Testing score 0–3 in any limb	7%	7%	8%	1.00^†^
Sensory loss	36%	36%	39%	0.86^**^
Aphasia	–	–	–	0.68^†^
None	84%	86%	77%	–
Mild	9%	7%	15%	–
Moderate	7%	7%	8%	–
Dysarthria	–	–	–	0.75^†^
None	82%	83%	77%	–
Mild/Mod	16%	14%	23%	–
Severe	2%	2%	0%	–
Ataxia	19%	19%	18%	1.00^†^
Neglect	7%	10%	0%	0.56^†^
Field Cut	6%	5%	8%	1.00^†^
Gait distance in feet, median (IQR)	50 (30–200)	80 (40–200)	40 (30–250)	0.78^‡^

*PTSD, post traumatic stress disorder; IQR, interquartile range*.

** *Chi-squared test*.

† *Fisher's exact test*.

‡ *Wilcoxon Rank Sum*.

Health-related outcomes are summarized in Table 5. Health care utilization was numerically higher in the group who met criteria for anxiety, depression and/or PTSD compared to those without, but this was not statistically significant (9 vs. 4, respectively, *p* = 0.06). There was no significant difference between these two groups in time from stroke to survey to account for differences in utilization (median 273 days vs. 278 days, *p* = 0.42). Thirty-two subjects reported working prior to their index stroke. Among these individuals, return to work was significantly less common in the group that had symptoms of mental health disorders than among those who did not [1 of 8 (13%) vs. 21 of 24 (88%), *p* < 0.01, Fisher's exact test].

**Table 5:** Outcomes after stroke among those with and without probable mental health disorder.

	**Overall**	**Depression, anxiety, and/or PTSD**		***P*-value**
		**Absent**	**Present**	
*N*	55	42	13	–
Health Utilization				
Physician visits, median	5	4	9	0.06^‡^
ED visits, median	0	0	0	0.44^‡^
Hospitalizations,	0	0	0	0.39^‡^
median				
Nights in the	0	0	0	0.36^‡^
hospital, median				
Good functional outcome (mRS 0–2)	86%	95%	54%	<0.01^†^
Euroqol: Subjects Reporting Any Problem				
Mobility	46%	33.3%	84.6%	<0.01^†^
Self Care	20%	9.5%	53.9%	<0.01^†^
Usual activities	47%	38.1%	76.9%	0.02^†^
Pain	53%	47.6%	69.2%	0.22^†^
Anxiety/depression	48%	21.4%	76.9%	<0.01^†^
Overall QOL Visual Analog Scale, median (IQR)	80 (60–90)	80 (60–90)	50 (50–60)	<0.01^‡^

*PTSD, post traumatic stress disorder; ED; Emergency department; mRS, modified Rankin Scale; QOL, quality of life; IQR, interquartile range*.

† *Fisher's exact test*.

‡ *Wilcoxon Rank Sum*.

Subjects with anxiety, depression, and/or PTSD reported significantly worse functional outcome compared to those without, as measured by the mRS (mRS 0–2: 54 vs. 95%, *p* < 0.01). Overall quality of life was also significantly lower in the group with symptoms of any mental health disorder compared to those without symptoms (median score of 50 vs. 80, *p* < 0.01). In a *post-hoc* exploratory regression model, the presence of a mental health disorder was associated with quality of life in the univariate model (β = −22.2, 95% CI: −33.0, −11.5, *p* < 0.01) and in a multivariable model including age, initial NIHSS, and mRS (β = −16.2, 95% CI: −28.9, −3.5, *p* = 0.01).

## Discussion

In this study of subjects with first-ever ischemic stroke and no prior psychiatric history, 24% of subjects had anxiety, depression, and/or PTSD 6–12 months after their stroke, with a high degree of overlap among these conditions. Further, subjects with these mental health disorders were less likely to return to work, had lower quality of life, and had worse self-reported functional status.

The differences in self-reported functional outcome and quality of life between those with and without anxiety, depression, and/or PTSD were large. Only 54% of subjects with anxiety, depression and/or PTSD reported good functional outcome (mRS 0–2) compared to 95% of subjects without these conditions. In the population who met criteria for these mental health disorders, overall quality of life was 30 points lower than those who did not (50 vs. 80, respectively). These differences are particularly striking given that the study population had predominately mild strokes and there were no measured differences in neurologic deficits. These data suggest that the presence of these mental health disorders may have a large impact on quality of life and functional outcomes after stroke.

Our study is not the first to report the association between mental health disorders and decreased functional outcomes. Paolucci et al. evaluated the relationship between post-stroke depression and functional outcomes, using the Barthel index and the mRS, as well as quality of life using the SF-36, and found that depressed subjects reported significantly higher rates of severe disability and decreased quality of life compared to non-depressed subjects (25). Naess et al. also used the SF-36 to assess health-related quality of life after stroke in young patients and showed that, in all items, scores were reduced in the group that screened positive for depression (38). Tse et al. also found that patients with mild symptoms, in this case mRS median 1, continued to have significant reduction in quality of life and in work and social engagement (39). These studies did not evaluate for differences in baseline NIHSS or neurologic symptoms between groups. Maaijwee et al. examined transient ischemic attack and ischemic stroke patients 10 years after stroke and found that anxiety and depression were associated with decreased functional outcomes (8). This study adjusted for baseline NIHSS; however, given the long time between the index event and the assessment of outcomes, there may have been other subsequent medical events that impacted functional status and outcomes. Chun et al. recently reported that 3 months after TIA or minor stroke health related quality of life and functional independence was worse in patients with anxiety, compared to those without anxiety, despite similar baseline severity. Consistent with our findings, they also reported large overlap between anxiety and depression (40).

Return to work was significantly less prevalent in the subjects that had anxiety, depression, and/or PTSD vs. those without these conditions. Previous studies have also looked at return to work in the post-stroke population. Hacket et al. found that 75% of patients returned to work with 12 months of stroke. Early depression was not associated with return to work in this population, but there was a trend toward not being depressed correlating with increased return to work and this study was likely underpowered to answer this question (41). Garrelfs et al. showed that the presence of psychiatric disorders was associated with decreased odds of returning to work in an acquired brain injury population, which included ischemic stroke, subarachnoid hemorrhage, and traumatic brain injury (42). A prior study by Tse et al. found that 56% of patients who were employed pre-stroke returned to paid employment post-stroke. This study did not look at the relationship between return to work and depression, but did note that returning to work and social activities was found to be independently associated with increased quality of life in multiple domains whereas depression was linked with decreased quality of life (39).

We also found a non-significant increase in health care utilization. Ghose et al. previously showed an association between depression and increased healthcare utilization after stroke (43). It is unknown whether preventing or treating psychiatric comorbidities after stroke could lower healthcare costs by improving return to work and/or reducing healthcare utilization, but this may be a fruitful area for further research.

We found that the PHQ-2 during admission was only positive in 50% of patients with depression at 6–12 months and 40% of patients with symptoms of depression, anxiety, or PTSD. The PHQ-2 has been previously validated for identifying concurrent depression in the post-stroke population, but these studies did not address the ability of the PHQ-2 in the acute setting to identify depression in the later post-stroke period (29, 30, 44). Optimal identification of post-stroke mental health disorders will likely require screening at multiple time points.

Identification of mental health disorders in the post-stroke population is not enough, as access to effective therapies is needed to prevent and/or treat these conditions. Studies have shown that selective serotonin reuptake inhibitors (SSRIs) and some serotonin-norepinephrine reuptake inhibitors (SNRIs) are safe and effective in this population and may help with both treatment and prevention of mental health disorders (45–47). Cognitive behavioral therapy (CBT) has also been shown to be effective for both prevention and treatment of post-stroke depression with limited data suggesting that CBT may provide more durable results than pharmacotherapy (47, 48). Combating social isolation and increasing activity participation have also been suggested as therapies for post-stroke depression (49, 50). There are numerous potential barriers to mental health treatments, including social stigma, concerns about polypharmacy, cost, and therapeutic inertia (51, 52). Access to traditional CBT may be particularly challenging for stroke patients who often have mobility limitations. Telehealth or other alternative delivery methods (e.g., through mobile technology) may be an option to improve access to CBT, and potentially at a lower cost, but more research is needed to confirm the efficacy of these alternative delivery methods after stroke (52–54).

## Limitations

There are several limitations to our study. Given the small sample size, we had limited power to explore differences or adjust for confounders in regression models. Non-response bias is possible given that subjects were informed of the topic of the surveys prior to consent and survey administration. Although responders and non-responders were similar on measured variables, if subjects with anxiety, depression, or PTSD were more or less likely to respond than other subjects, the rates of these conditions reported in our study may be biased. Additionally, because we used medical record review and self-reporting to identify pre-stroke psychiatric disorders, it is possible that patients were included who had pre-existing undiagnosed psychiatric disorders. Despite these limitations, the rates of depression, anxiety, and PTSD in our study are similar to other published studies in patients with stroke (5, 7–12). Although we utilized validated screening tools to define anxiety, depression, and PTSD, these tools do not equate to a clinical diagnosis. We excluded patients who were aphasic or were discharged to a skilled nursing facility, as well as patients with preexisting psychiatric disease, so our results may not be generalizable to all stroke patients. Given the hypothesis generating nature of this study, we did not correct for multiple comparisons, which could increase the risk for type I error. Finally, based on our study design, we are unable to assess the causality of the observed relationships between these mental health disorders and outcomes. Future longitudinal studies would be helpful in this regard.

## Conclusions

Post-stroke psychiatric disorders are common, and there is significant overlap in anxiety, depression, and PTSD. Subjects with these disorders had lower functional status, worse quality of life, and were less likely to return to work, despite having similar baseline stroke severity and neurologic deficits. Further elucidating the relationship between anxiety, depression, and PTSD and post-stroke outcomes, specifically QOL, healthcare utilization, and return to work, and identifying effective, accessible methods to prevent and/or treat post-stroke mental health disorders should be a high priority.

## Ethics statement

This study was carried out in accordance with the recommendations of the Hospital of the University of Pennsylvania Institutional Review Board with written informed consent from all subjects. All subjects gave written informed consent in accordance with the Declaration of Helsinki. The protocol was approved by the Hospital of the University of Pennsylvania Institutional Review Board.

## Author contributions

LS: project proposal and design, data gathering (phone calls, chart reviews, etc.), data entry, and manuscript writing; EG: project proposal and design, contribution largely based on stroke and psychiatric epidemiology experience, and manuscript revisions; AZ: data gathering (phone calls, chart review, etc.), data entry, and manuscript revision; JL: project development assistance and manuscript revisions; SM: project development and manuscript revision; BC: project development and manuscript revision; SK: project development and manuscript revision; MM: project development, project oversight, data analysis, manuscript review and revision.

### Conflict of interest statement

The authors declare that the research was conducted in the absence of any commercial or financial relationships that could be construed as a potential conflict of interest.
