# Supplementary material for: Association Between Anxiety, Depression, and Post-traumatic Stress Disorder and Outcomes After Ischemic Stroke
Source: Front Neurol. 2018 Nov 2;9:890. doi: 10.3389/fneur.2018.00890 (PMC6224432; doi:10.3389/fneur.2018.00890)
Supplement: Supplementary file 1 [file Data_Sheet_1.docx]

**Phone Interview**

*I’m going to start off with a few questions about your life.*

What do you consider your race to be? (White, Black, Asian or Pacific Islander, Other) ______________

What do you consider your ethnicity to be? (Hispanic, non-Hispanic) ______________

What level of education did you complete? (Did not graduate high school, high school degree, college degree, masters/PhD/other advanced degree) ______________

Prior to your stroke, had you ever been diagnosed or treated for a psychiatric disease? (yes/no) ______________

If yes, what psychiatric disease? (Free text) ______________

Were you working prior to your stroke? (yes/no) ______________

Are you working now? (yes/no) ______________

If not, what is the limitation to returning to work? (Free text) ______________

Counting yourself, how many members currently make up your household? (Integer) ______________

Are you now married, widowed, divorced, separated, never married, or living with a partner? (married, widowed, divorced, separated, never married, living with partner, refused) ______________

Did you go to inpatient rehabilitation after your stroke? (yes/no) _______________

If so, how long were you in inpatient rehabilitation? (in days, integer) _______________

*Next, I will ask you a few questions about how often you have received medical attention in the past 6 months. Do NOT count your initial stroke hospitalization or rehab stay:*

**Stanford Healthcare Utilization Survey**

1. In the past 6 months, how many times did you visit a physician? Do **not** include visits while in the hospital or to a hospital emergency room. Fill in with “0” or another number.
2. In the past 6 months, how many times did you go to a **hospital** emergency room? Fill in with “0” or another number.
3. How many different **times** did you stay in a hospital **overnight** or longer in the past 6 months? Fill in with “0” or another number.
4. How many total **nights** did you spend in the hospital in the past 6 months? Fill in with “0” or another number.

Have you been told by a doctor that you had a second stroke separate from your first stroke? ________

*Next, I would like to ask you some questions about how you have been feeling during the past week. It is best not to think too hard about your response, but to give the answer that first comes to mind.*

**Hospital Anxiety and Depression Scale**

[
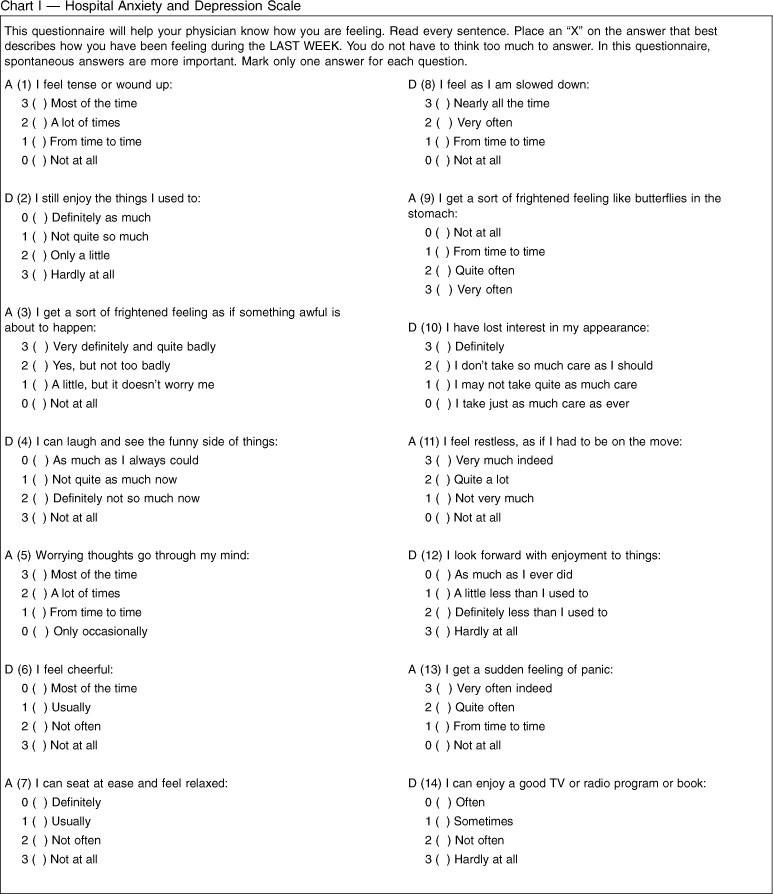
](http://www.google.com/url?sa=i&rct=j&q=&esrc=s&source=images&cd=&ved=2ahUKEwiPzonB2MLdAhXNct8KHbTDC_kQjRx6BAgBEAU&url=http://www.scielo.br/scielo.php?pid%3DS0034-70942007000200004%26script%3Dsci_arttext%26tlng%3Den&psig=AOvVaw1uC6mD5fMgUf9P_UREz_O1&ust=1537296130983689)

*We will now read you a list of problems and complaints that some people sometimes have in response to stressful life experiences, like a stroke. Please indicate how much you have been bothered by that problem in the last month by giving a number on a scale of 1-5 where 1 is not at all, 2 is a little bit, 3 is moderately, 4 is quite a bit, and 5 is extremely.*

**PTSD CheckList- Stressor Specific Version (PCL-5)**

The event you experienced was: ___________________ on: ____________________


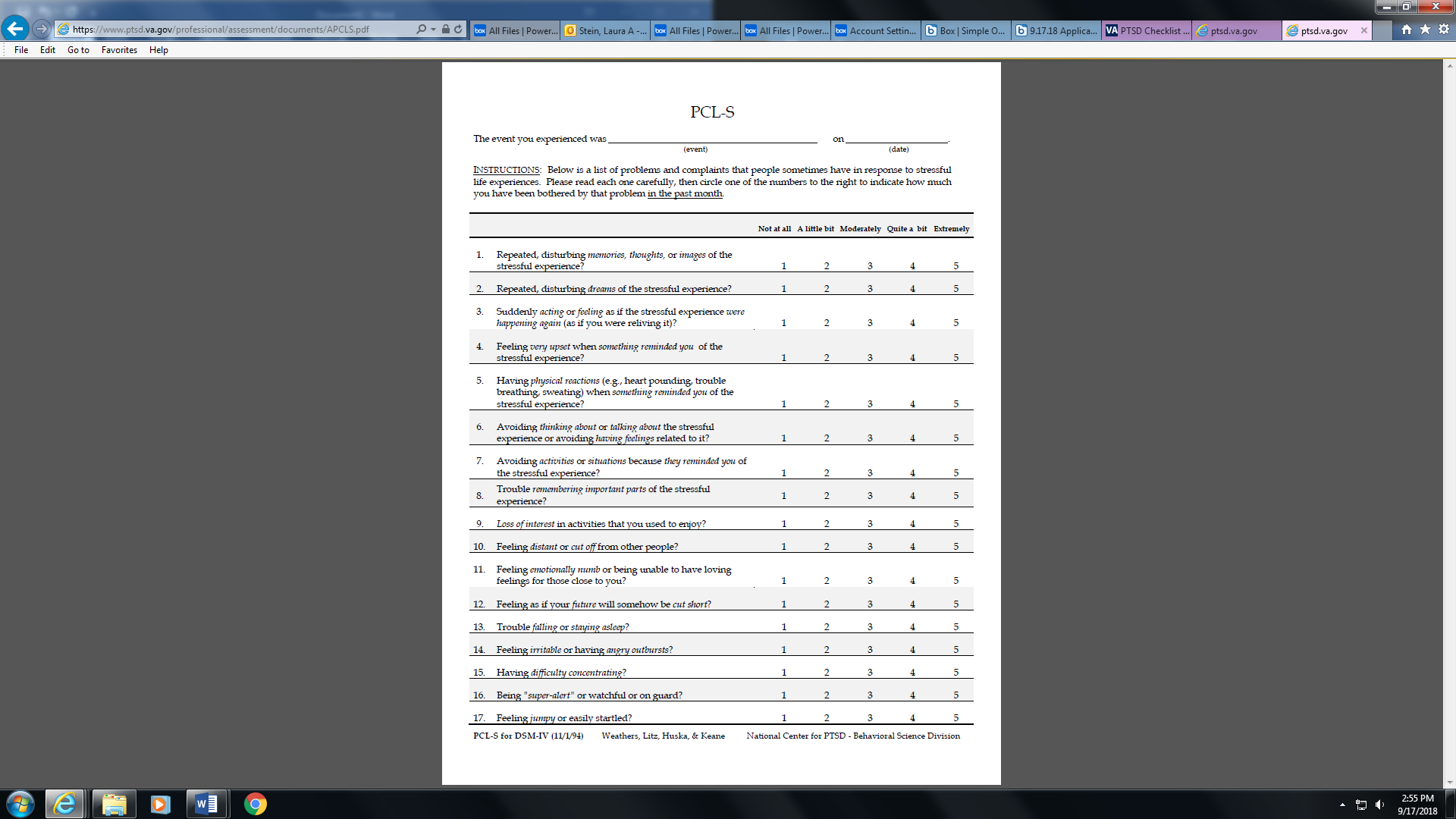


Positive/Negative: ________ Score: _________

*I would also like to know how you are functioning after your stroke. Please let me know which of the following scenarios best describes your level of function at this point.*

**The Modified Rankin Scale & Corresponding Sections of Structured Interview**

[
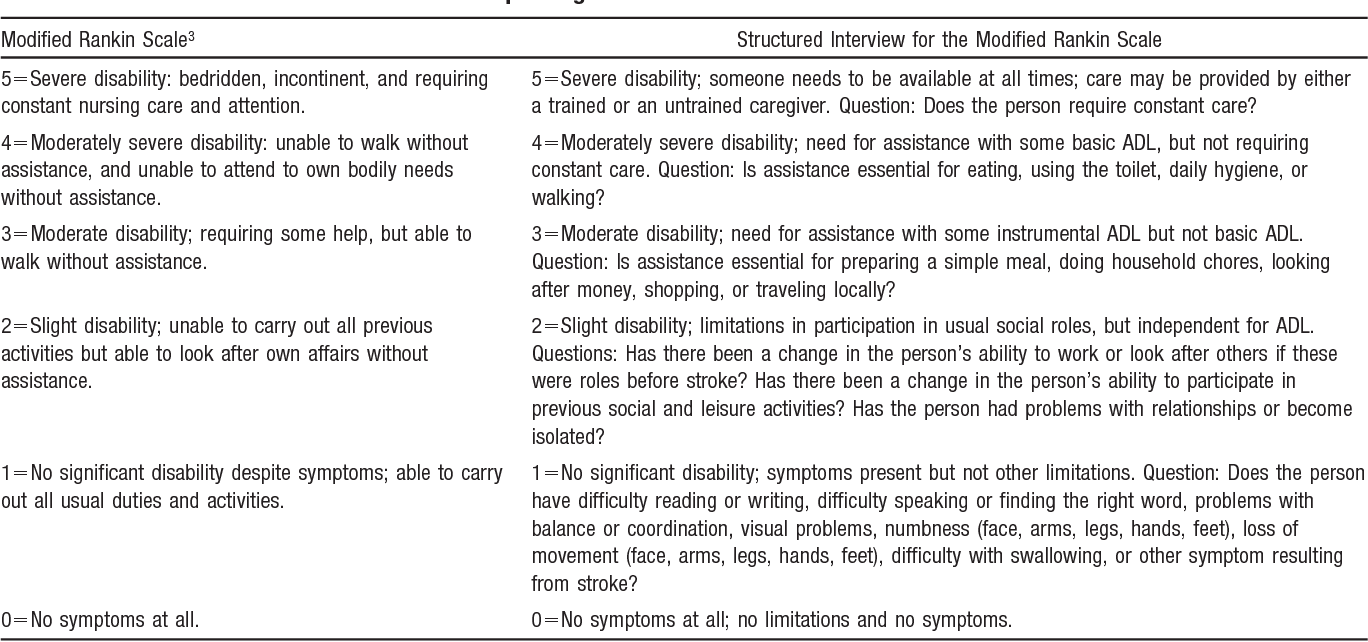
](https://www.google.com/url?sa=i&rct=j&q=&esrc=s&source=images&cd=&cad=rja&uact=8&ved=2ahUKEwjNmeaB38LdAhWI3lMKHXNmDH0QjRx6BAgBEAU&url=https://www.semanticscholar.org/paper/Improving-the-assessment-of-outcomes-in-stroke%3A-use-Wilson-Hareendran/8ce8cedd770a0b06b38b33ed83feeab638dc5893&psig=AOvVaw0WZl7dfVkPKKxLdUncfjUP&ust=1537297875533715)

*Please indicate which statements best describe your own health state today.*

**Euro QOL**

**Mobility**

___ I have no problems in walking about

___ I have some problems in walking about

___ I am confined to bed

**Self-Care**

___ I have no problems with self-care

___ I have some problems washing or dressing myself

___ I am unable to was or dress myself

**Usual Activities** *(e.g. work, study, housework, family or leisure activities)*

___ I have no problems with performing my usual activities

___ I have some problems with performing my usual activities

___ I am unable to perform my usual activities

**Pain/Discomfort**

___ I have no pain or discomfort

___ I have moderate pain or discomfort

___ I have extreme pain or discomfort

**Anxiety/Depression**

___ I am not anxious or depressed

___ I am moderately anxious or depressed

___ I am extremely anxious or depressed

To help people say how good or bad a health state is, we have produced a scale (rather like a thermometer) on which the best state you can imagine is marked **100** and the worst state you can imagine is marked **0**.

We would like you to indicate on this scale how good or bad your own health is today, in your opinion. Please pick a number between 0 and 100 that best describes your health state.

Health state: ____________
